# What Should Be Our Ideal in Prosthetics

**Published:** 1922-08

**Authors:** W. F. Chappelle

**Affiliations:** Buffalo. N. Y.


### WHAT SHOULD BE OUR IDEAL IN PROSTHETICS

BY W. F. CHAPPELLE, D.D.S., BUFFALO. N. Y.

Denture construction has not seemed to keep pace with other branches of dentistry in that there has been little effort to standardize the procedure. The work, as usually practiced, is given little importance, is done in a haphazard manner and the results are largely a matter of luck. Very little effort is made to study arch and tissue formation, esthetic results or masticating efficiency. Practically all cases receive the same general treatment or lack of it.

Among the comparatively few who are trying to do real work, a wide diversity of opinion exists as to the methods used and results desired; and no real effort has been made to bring them together and standardize the method. A great deal of hard faithful work has been done by such men as Gritman, Snow, Bonwill, Gysi, Hall, Monson, Needles, Hanau, the Green Brothers, and Tench, and it seems that a more rapid development should follow. I shall not mention names further in order to keep the personal element out, but shall deal simply with the methods these men have advocated, as they have all helped wonderfully to stimulate better prosthetic processes.

With regard to impression taking, red compound alone, black compound and plaster, a base plate and wax, and plaster alone are most prominent. Each has its place and we should be familiar with them all and be able to use them where indicated. No single one will meet every requirement, although those who are less skilled can perhaps develop one method of impression taking and get better results for average cases. The red compound, as ordinarily used, is best suited for getting an impression under pressure; the black compound and plaster, when used without excessive valve-seal and post-damming, gives minute detail with little pressure. The base plates and waxes of different melting-points give a very easy-feeling impression valuable for sore and tender mouths. These three methods all allow accurate muscle trimming. Plaster alone can be varied greatly according to the consistence at which it is placed in the mouth, but it is hard to muscle-trim. All agree that excessive valve-seal and post-damming should be avoided because they interfere with the circulation.

So far as esthetics are concerned, proper length and mold of teeth, proper overbite and carving, properly selected and bleached pink rubber and correct shaping of palatal surfaces of teeth and denture, all add greatly to the patient's comfort and appearance, help the speech and ease self-consciousness in possessing denture; all being important considerations, but if neglected do no great harm to the tissues involved or to the general health of the patient.

The most important consideration and the one where, unfortunately, we are most widely divided, is the articulation. On this important point those who give it prominence are divided into three groups:

First, those who advocate articulators, working in accordance with arbitrarily accepted symmetrical, geometrical designs, as a cone, sphere or concentric spheres. Some recommend the use of a facebow, but as a rule they do not teach its use as absolutely essential, as they favor the floating condyle theory.

Second, those who use articulators accepting in general average dimensions and movements of the masticatory apparatus, incorporating these in a non-adjustable articulating mechanism and using a facebow.

Third, those who use an adjustable articulator and insist on a face-bow registration and transfer.

The first group pays no attention to the magnitude and angle of the condyle paths so far as measuring them and coördinating cusp angles, incisor guide and curve of Spee to these measurements, but give a balanced bite on an arbitrary articulation and claim that the patient can adapt himself to the dentures. They rely upon accommodating tissue changes, the so-called floating condyle theory and even expect permanent changes in the temporo-mandibular apparatus to accommodate the denture articulation. All these assumptions are reinforced by the claim that the teeth guide the condyles instead of the condyles guiding the teeth.

The second group uses an average condyle inclination approximating actual mandibular movements and gives a balance bite on an articulation that fits these articulators, and then depends upon the use of carborundum and vaselin in the mouth to complete the adaptation.

The third group measures the actual magnitude and inclination of the more important mandibular movements and works out an articulation that will be as good or better than the natural articulation, will co-ordinate accurately with those movements and will not interfere with their free and natural performance.

The second and third groups differ only in the closeness of approximating an ideal articulation. So far as esthetic results and maximum efficiency are concerned, they fall short.

We should now consider prosthetics from another angle. Should a longer time be needed for making dentures correctly, or a lack of skill necessary in certain methods, through unwillingness or inability to study those methods, have consideration in deciding which is the ideal method? Have we a right to cause our patient's discomfort if avoidable? Is it right to cause changes in the mouth tissues or in the temporo-mandibular articulation if unnecessary? If we limit or otherwise reduce maximum masticating efficiency, are we conserving the digestive apparatus of our paients? Some of us may not see fit to allow these things to influence our actual practice, but they should certainly be very important in choosing between perfect and imperfect methods and results.

In comparison, let us take group I and group III, since group II is similar to III, but does not go as far in its efforts to copy natural and free anatomic conditions.

The grouping of methods referred to in group I, may be a little radical, but the idea is to call attention to the tendency of the advocates of these methods to depart from natural conditions and to minimize the importance of actual measurement and coordination with the important condyle paths.

*Group I.* (1) If no attempt to establish relative positions of arches and condyle centers or axes is made, any position outside of central occlusion may result in an unbalanced bite due to the divergence of two radii at different distances from the common center.

(2) Necessity of limited overbite and curve of Spee in most dentures, due to the danger of not balancing when actual inclination of condyle path is unknown, providing free mandibular movements are made and granting that the condyles guide the teeth.

(3) Necessity of symmetric articulation due to inability to adjust sides of articulator for asymmetric condyle paths.

(4) Resulting in lack of ability to give complete consideration to esthetics due to point 2.

(5) Resulting in lack of stability, necessitating greater suction and consequent interference with circulation due to points 1 and 3.

(6) Limiting of mandibular movements and impairment of masticatory efficiency due to the first three points.

(7) Soreness and discomfort to patient through incoordination with natural movements and necessity of learning new movements.

(8) Greater absorption of ridges due to instability and unequal pressure on ridges.

(9) Most serious of all is the inviting of changes in the temporo-mandibular articulation due to either excessive pressure or lack of contact on the interarticular cartilages and bone beneath, because of reliance on the theory that the teeth guide the condyles and not the condyles guide the teeth, which together with tissue compressibility is called the floating condyle.

*Group III.* Here we have almost opposite conditions:

(1) The facebow gives the positional relation of arches to the condyle centers so that the relative distances front and back teeth open, when dentures are out of central occlusion, as represented by divergence of radii at varying distances from center, are always the same as in the mouth.

(2) Actual condyle path inclination and lateral path inclination, also the influence of other momentary axes of rotation due for instance to both condyles simultaneously making antero-posterior excursions in opposite directions outside of the glenoid fossæ, are determined closely enough so that error is negligible.

(3) Knowing always the two condyle paths, or at least two corresponding momentary centers of rotation, an arbitrary third point, an incisor guide of the desired angle to give ideal esthetic results or to increase or decrease cusp angles for maximum masticatory efficiency and stability, can be chosen; due to the fact that compensating angles will result at any point between incisor guide and condyle path angles.

These points result in—

(4) No limit to esthetic results.

(5) Greater stability in any relative position of the dentures due to asymmetrical articulation, and perfect balance and coordination of cusps with free and natural masticatory movements.

(6) No limitation of freedom of movement.

(7) Much less soreness and discomfort due to this coordination.

(8) Much less absorption of tissues, as dentures are stable.

(9) No changes needed in temporo-mandibular articulation. Teeth guided by condyles as in natural mechanism.

The use of carborundum in the mouth to the extent it is usually advocated except possibly in extreme cases is, we believe, decidedly a poor expedient. It simply covers the faults of the articulator to the detriment of cusp angles and efficiency.

So far as disproving the theory of teeth guiding the condyles and not the condyles guiding the teeth, we are reminded of a point made by one of the investigators mentioned, that if the teeth guide the condyles readily, then there should be no such condition as traumatic occlusion as applied to a considerable number of teeth, and no discomfort from a badly articulated bridge necessitating relief by grinding. If not relieved, malocclusion results in rapid changes in the tissues involved, if severe enough; or slow progressive permanent changes in the tissues surrounding the teeth, if less severe. We can never expect adjustment of condyles to the malocclusion as readily as would be desired to make the condyle theory an ideal one to take advantage of. The greater severity of disturbance in the ridges and the condyle region with a case of severe malocclusion than when a set of artificial dentures is present, is explained by the fact that natural teeth cannot give as readily as the artificial dentures can raise from or compress the mucosa and cause bone to absorb under unequal pressure in the mouth, and also to the fact that the patient with natural dentures still is familiar with his natural free mandibular movements and insists on using them. With dentures, he is more apt to limit them if he thinks it is necessary. He also limits the masticatory pressure exerted due to tenderness of the tissues and hence has less displacement until tissue changes have time to result.

The more a patient limits himself to a chop bite into central occlusion the less trouble he gets into.

I have tried to make this article anything but technical, have avoided diagrams and the mention of geometry, statics, or kinematics. I have made it impersonal, for we are all striving for the best and any research work influences and aids a profession greatly, but it seems as though such wide divergence is unnecessary, and out of so much hard work we should be able to evolve an ideal so that the younger men and the students at least should have a standard which might be developed to perfection. Too many different methods are taught and they are too far apart. Universal centers of rotation cannot be momentary and permanent as well. An attempt is made to make us believe that universal or semi-universal centers or axes of rotation are located by one theory above, by another below, by still another far posterior to the mandible. This very extreme divergence would strongly indicate serious deficiency in the methods of research. If there is one ideal method and the others have any merits as expedients, they should be rated as such and their limits emphasized, but all should not be on an equal basis, at least so taught in colleges.—*Dental Cosmos.*

